# Rapid Nondestructive Detection of Water Content and Granulation in Postharvest “Shatian” Pomelo Using Visible/Near-Infrared Spectroscopy

**DOI:** 10.3390/bios10040041

**Published:** 2020-04-20

**Authors:** Sai Xu, Huazhong Lu, Christopher Ference, Guangjun Qiu, Xin Liang

**Affiliations:** 1 Public Monitoring Center for Agro-Product of Guangdong Academy of Agricultural Sciences, Guangzhou 510640, China; xusai@gdaas.cn (S.X.); qiuq16@scau.edu.cn (G.Q.); L13060900589@163.com (X.L.); 2 Guangdong Academy of Agricultural Sciences, Guangzhou 510640, China; 3 Department of Plant Pathology, University of Florida, 2550 Hull Road, Gainesville, FL 32611, USA; cference@ufl.edu

**Keywords:** visible/near infrared spectroscopy, pomelo, granulation, water content, detection

## Abstract

Visible/near-infrared (VIS/NIR) spectroscopy is a powerful tool for rapid, nondestructive fruit quality detection. This technology has been widely applied for quality detection of small, thin-peeled fruit, though less so for large, thick-peeled fruit due to a weak spectral signal resulting in a reduction of accuracy. More modeling work should be focused on solving this problem. “Shatian” pomelo is a traditional Chinese large, thick-peeled fruit, and granulation and water loss are two major internal quality factors that influence its storage quality. However, there is no efficient, nondestructive detection method for measuring these factors. Thus, the VIS/NIR spectral signal detection of 120 pomelo samples during storage was performed. Information mining (singular sample elimination, data processing, feature extraction) and modeling were performed in different ways to construct the optimal method for achieving an accurate detection. Our results showed that the water content of postharvest pomelo was optimally detected using the Savitzky–Golay method (SG) plus the multiplicative scatter correction method (MSC) for data processing, genetic algorithm (GA) for feature extraction, and partial least squares regression (PLSR) for modeling (the coefficient of determination and root mean squared error of the validation set were 0.712 and 0.0488, respectively). Granulation degree was best detected using SG for data processing and PLSR for modeling (the detection accuracy of the validation set was 100%). Additionally, our research showed a weak relationship between the pomelo water content and granulation degree, which provided a reference for the existing debates. Therefore, our results demonstrated that VIS/NIR combined with optimal information mining and modeling methodswas feasible for determining the water content and granulation degree of postharvest pomelo, and for providing references for the nondestructive internal quality detection of other large, thick-peeled fruits.

## 1. Introduction

Pomelo (*Citrus maxima* (Burm) Merr.) is a traditional Chinese fruit that has been cultivated for thousands of years. Today, it is mainly cultivated in several countries around the world, including China, America, and Thailand, with a global annual yield of over 10 million tons, and the total cultivated area and annual yield are increasing [1]. The “Shatian” pomelo is one of the most favored pomelo cultivars in the world and has a relatively high sweetness. “Shatian” is usually harvested in December, then stored and served throughout the year. Previous research reported that granulation of the fresh inside was a common physiological disorder for postharvest “Shatian” pomelo during storage [2,3]. The granulation of pomelo is due to lignification results in the inedible flesh of a lighter color, along with the changed water content [4]. Thus, it is vital to the pomelo industry to be able to rapidly and nondestructively detect and monitor the water content and granulation degree of postharvest “Shatian” pomelo during storage.

Unlike small and thin-peeled fruits, the internal quality of pomelo is difficult to discern due to its thick peel and large size. Additionally, no matter how much the internal quality degrades, the outside appearance will barely change, which makes the internal quality of postharvest pomelo almost impossible to determine by visually inspecting the fruit [5]. With an increase in consumer standards, guaranteeing product quality is vital for the fruit industry. Combined with its low peelability, a high incidence rate of inferior pomelo making it to the market would erode consumer acceptance, creating a bottleneck for pomelo industrial development.

Traditional pomelo internal quality detection methods include sensory [6] and physicochemical index methods [7,8], both of which are time- and labor-intensive, damage and waste fruit, and cannot be applied to detect and guarantee the quality of every postharvest pomelo. Machine vision has been widely applied as a fast, intelligent, and nondestructive method for grading fruit quality [9,10], but this only provides information concerning external characteristics.

Visible/near-infrared (VIS/NIR) spectroscopy has been increasingly applied for quality detection for fruit, such as apple, orange, peach, and kiwi, often combined with fruit-specific calibration models for fast and accurate measurement [11,12,13,14,15]. The absorbance of NIR is mainly affected by the stretched vibration overtones and combination modes of hydrogen-containing groups (X–H) including O–H, N–H, C–H, and S–H [16]. Hence, NIR is sensitive to water content (H_2_O) differences. Pomelo granulation coincides with flesh color and a density change. VIS is primarily sensitive to color [17], and VIS/NIR was shown to be able to detect fruit density [18]. Existing research results informed the methods for this study. However, most VIS/NIR spectroscopy applications are for small, thin-peeled fruit, instead of large, thick-peeled fruit like pomelo. A large size or thick peel can result in a weak VIS/NIR signal with increased noise, which results in a poor internal quality detection accuracy. To solve this problem, previous studies showed that it is feasible to measure internal melon quality using VIS/NIR spectroscopy by transmitting light from the side of the fruit to the bottom [19,20]. However, our research found that the spectral signal transmissivity and anti-noise performance of pomelo detection is even worse than melon, mainly due to the pomelo pericarp including a mass of oil vacuoles and a thick spongy mesocarp. Additionally, the higher density and internal complexity of pomelo relative to a melon also results in increased difficulty regarding accurate internal quality detection. Further advancement of the internal quality detection of pomelo fruit is still needed to find an optimal modeling and detection method.

Thus, this research aimed to develop a method and statistical procedure to efficiently measure the internal water content and granulation of postharvest pomelo. The VIS/NIR spectrum signal characteristics of postharvest pomelos were collected and investigated. The singular sample elimination, data processing, feature extraction, and modeling work were performed in different ways to find the optimal procedure to measure pomelo internal water content and granulation. Additionally, the relationship between water content and granulation was analyzed.

## 2. Materials and Methods

### 2.1. Experimental “Shatian”Pomelo

“Shatian” pomelo fruits, 120 in total, were harvested from an orchard in Meizhou city, Guangdong province, China, then shipped to our lab in Guangzhou city within 24 h and stored at room temperature. As granulation often occurs approximately 3 months after harvesting [21], two stored pomelo fruit were sampled every other day between 3 and 5 months of storage.

### 2.2. VIS/NIR Spectrum Sampling

**Figure 1:**
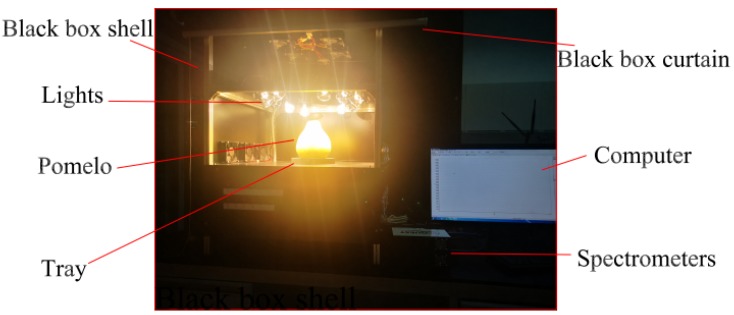
Structure of the lab-developed VIS/NIR spectrum sampling platform.

Our lab developed a VIS/NIR spectrum sampling platform, as shown in Figure 1. To reduce the external light, pomelo samples were measured in a dark box. There were two sets of lights, one on each side, with each set including six 100W halogen lamps. In consideration of the practical needs of an assembly line detection, a movable tray was applied to convey and stabilize each tested pomelo. The spectrum signal transmitted through the pomelo was translated into a digital signal using two spectrometers (QE PRO with wavelengths of 400–1100 nm and NIR QUEST with wavelengths of 900–1700 nm, Ocean Optics Inc., Dunedin, FL, USA). Thus, the transmitted spectrum wavelengths between 400 and 1700 nm could be tested and recorded. For sampling, the pomelo fruit were aligned stem-end up on the tray, with spectrum signal wavelengths of 400–1050 and 1050–1700 nm acquired by the QE PRO and the NIR QUEST spectrometers, respectively. The pre-sampling process was: (1) save the dark current value D, (2) offset the dark current value (D of NIR QUEST plus the difference between D from the QE PRO and D from the NIR QUEST at 1050 nm), and (3)save the reference value R (3.6 cm thick spectral calibrated panelmade of barium sulfatematerial). Finally, with the pomelo sampling detector response value (P), the pomelo transmissivity was equal to (P − D)/(R − D).

### 2.3. Water Content and Granulation Degree Test

**Figure 2:**
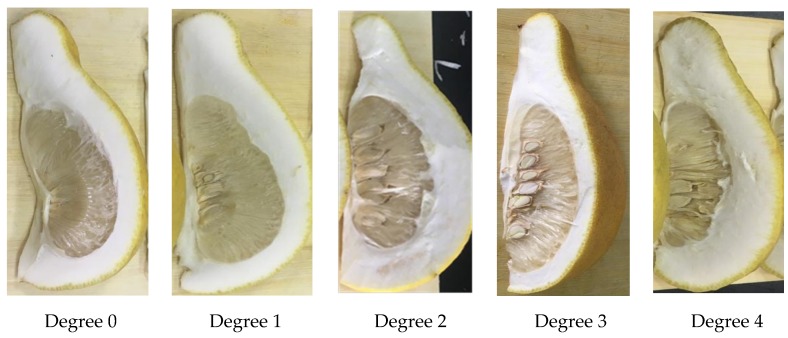
Pomelo sections with different granulation degrees.

The water content and granulation degree assessment was conducted after the VIS/NIR spectrum sampling. In accordance with previous research [22], the granulation degree was tested by cutting pomelo fruits into 1/8 lengthways, and the ratio between the granulation area and the total fruit area was measured. A granulation degree of 0 means the ratio was 0%, a granulation degree of 1 means the ratio was between 0% and 10%, a granulation degree of 2 means the ratio was between 10% and 25%, a granulation degree of 3 means the ratio was between 25% and 40%, and a granulation degree of 4 means the ratio was greater than 40% (Figure 2). Then, 200g of flesh was taken from eight pomelo pieces and put in an oven set to 60 °C for 24 h. The water content percent was calculated using ((fresh weight before drying − fresh weight after drying)/fresh weight before drying) × 100.

### 2.4. Data Analysis Methods

#### 2.4.1. Singular Sample Removal

Clusteringanalysis (CA) [23], which is the task of grouping a set of objects in such a way that objects in the same group (called a cluster) are more similar to each other than to those in other groups, was applied to identify singular samples.

#### 2.4.2. Data Preprocessing

Data processing is an efficient way to magnify the differences among samples to improve the detection accuracy of the pomelo water content.Thus, six commonly used data processing methods—the first derived method (FD), squareroot method (SR), logarithm method (LM), inverse method (IM), Savitzky–Golay method (SG), and multiplicative scatter correction method (MSC)—were each used to process spectral data to determine the optimal one [24,25]. The aims of the FD, SR, and LM methods are to show the data from different angles using first-order differential, square root, and logarithm algorithmsbytransforming the raw spectral data. Thus, these methods have the potential to help with detection, particularly when sample differences cannot be easily discerned from the raw data. SG and MSC are both used for noise reduction. SG can be applied to a set ofdigital datapoints to smooththe data by increasingthe data precision without distorting the signal tendency. MSC aims toalignthe spectra data in such a way that they are as close as possible to a reference spectrum and the mean of the data set by changing the scale and the offset of the spectra. For the SG operation, the polynomial order and the frame length (smooth points) are key parameters that influence the processing method.

#### 2.4.3. Feature Selection

The regression coefficient algorithm (RCA), mutual information–successive projections algorithm (MI-SPA), genetic algorithm (GA), and principal component analysis (PCA) were applied to select the features for modeling [26,27,28]. Regressioncoefficients were applied to describe the relationship between a predictor variable and the output value, where the variable with a higher regressioncoefficient has a stronger relationship with the output value. SPA is a forward selection method that uses simple operations in a vector space to minimize variable co-linearity. Concerning the MI-SPA, the MI selects the feature (the single spectral feature with the highest MI value for the target output) that the SPA operation assigns as a reference. GAs are commonly used to generate high-quality solutions for optimization and search problems by relying on biologically inspired operators, such as mutation, crossover, and selection, and thus for GA operation, the initial population number, the crossover probability, the mutation probability, and the number of iterations are all key parameters that influence the feature selection method. PCA uses an orthogonal transformation to convert a set of observations of possibly correlated variables into a set of values of linearly uncorrelated variables called principal components.The principal component with a higher factor score contains more sample information.

#### 2.4.4. Classification and Detection

Linear discriminant analysis (LDA) [29], a method used inpattern recognition to find alinear combinationoffeaturesthat characterizes or separates two or more classes of objects or events, was applied to check the VIS/NIR-based granulation degree classification ability.

Partial least squares regression (PLSR) [30] was used to construct a pomelo water content and granulation degree detection model that can show the effect of singular sample removal, data preprocessing, feature selection, and build up the optimal detection model. PLSR finds a regression model by projecting the predicted variables and the observable variables to a new space, which is a popular modeling method due to its relatively good detection performance and fast operating rate. The coefficient of determination (R^2^) is the key parameter for evaluating the correlation between the predicted value and the actual value. The range of R^2^ is from 0 to 1, where a greater R^2^ equals a better predictive ability (a stronger relationship between the predicted value and the actual value). Additionally, the root mean squared error (RMSE) is another way to evaluate a detection method; the closer the RSME value is to 0, the better the method’s prediction (i.e., a smaller error between the predicted value and the actual value).Thus, theaccuracy assessment of water content detectionused the R^2^ and RMSE of the validation set. However, the granulation degree detection is a classification problem, and evaluating the rate of accurate and inaccurateresults is more appropriate for measuringthe detection accuracy than R^2^ and RMSE. Thus, for granulation detection, the integer of the PLSR output wasused to assign the granulation degree category.

#### 2.4.5. Analysis Parameter Setting

In this study, at the beginning of the PLSR water content detection, the entire sample population of postharvest pomelo fruits (labeled 1 to 120) were randomly divided into calibration and validation sets of 90 and 30 elements, respectively. After a singular sample elimination, 90 and 29 samples of calibration and validation sets were rested for the next water content modeling work. For the granulation PLSR detection, 9, 8, 13, 21, and 30 samples (about two-thirds of samples in each granulation degree group) were selected randomly from degree 0 to 4, respectively, as the calibration set (81 samples in total), with the remaining 38 samples comprising the validation set.After repeated detection model runs were performed to choose the optimal parameters, fourth-order seven-point SG and third-order five-point SG were applied for the data processing of water content and granulation detection, respectively. The parameters of GA for the water content detection spectral data in this study were: an initial population number of 70, a crossover probability of 0.5, a mutation probability equal to 0.01, and 120 iterations. Feature extraction was not run for the granulation detection spectral data to sample the operation process due to the detection effect already being satisfied.The optimal variable factor number forthe PLSR models were all 18. RCA, GA, MI-SPA, PCA, and PLSR can rank all feature variables from most to least important.The parameterswere selected by gradually increasing the feature variability according to the rank until the R^2^ of the validation set stopped increasing and the RMSE of the validation set stopped decreasing.

#### 2.4.6. Software Applied

All data analysis was performed using Matlab R2017a software (MathWorks Inc., Natick, MA, USA).

## 3. Results and Discussion

### 3.1. Water Content Detection

#### 3.1.1. Detection Based on Raw Data

**Figure 3:**
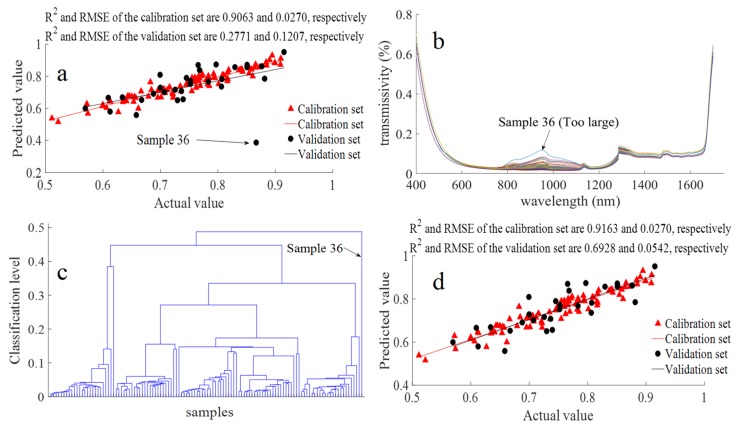
Pomelo water content detection based on the spectral raw data and singular sample elimination: (**a**) partial least squares regression (PLSR) analysis result based on all data, (**b**) differences betweenthe raw data of all samples, (**c**) singular sample discovery based on Euclidean distance clustering, and (**d**) PLSR analysis resultbased on singular sample eliminated data.

The PLSR analysis was performed as a preliminary check of the VIS/NIR spectrum raw-data-based water content detection method. The R^2^ and RMSE values of the calibration and validation sets for PLSR water content detection based on the raw data are shown in Figure 3a. The R^2^ and RMSE of the validation set were 0.2771 and 0.1207, which is a poor detection response (R^2^ less than 0.3). However, a data point from the validation set (sample 36) was an outlier and could be treated as a singular sample. The VIS/NIR spectrum raw data curve of all samples (Figure 3b) shows that the transmissivity curve of sample 36 was notably different from other samples, particularly between wavelengths 800 to 1150 nm. It is worth noting that the step (sharp increase) around 1300 nm should be a normal feature rather than an offset according to our previous research results (there was a step at 1000 nm in thetea spectral data but no step at 1000 nmfor the guava spectral data usingthe same spectral detector) [31,32], and 1300 nm is not the joint of the sampling data of spectral detectors in this study. The reason may be that the 750–1300 nm range includes weak water absorption bands, but the 1300–1600 nm range includes strong water absorption bands. The clustering analysis result from the raw VIS/NIR spectrum data of all 120 samples (Figure 3c) further shows the inference that sample 36 was a singular sample that could not be classified with all other samples. Thus, sample 36 was eliminated from the next analysis. After this single sample elimination, the PLSR results showed that the water content of pomelo could be reliably detected using VIS/NIR spectroscopy (Figure 3d); however, the R^2^ value of the validation set was 0.6928, which could be further improved. Thus, the singular sample elimination was a very important step, especially for large, thick-peeled fruit, where the difference among samples was larger than for small, thin-peeled fruit.

#### 3.1.2. Detection Based on Data Processing Methods

The PLSR detection results were combined with the six data processing methods (FD, SR, LM, IM, SG, and MSC), as shown in Table 1. Compared with the PLSR detection results based on raw data (RD), only the fourth-order seven-point SG and MSC improved the PLSR water content detection results, with increases in the R^2^ value for the validation set from 0.6928 to 0.7053 and 0.6998, respectively, anddecreases in the RMSE value of the validation set from 0.0542 to 0.0527 and 0.0500, respectively. Other data processing methods reduced the detection accuracy. Even the SG and MSC processes slightly decreased the R^2^ and RMSE before calibration; however, the PLSR result of their calibration set performed well and the PLSR result of the validation set was the result that reflected the detection performance.

**Figure 4:**
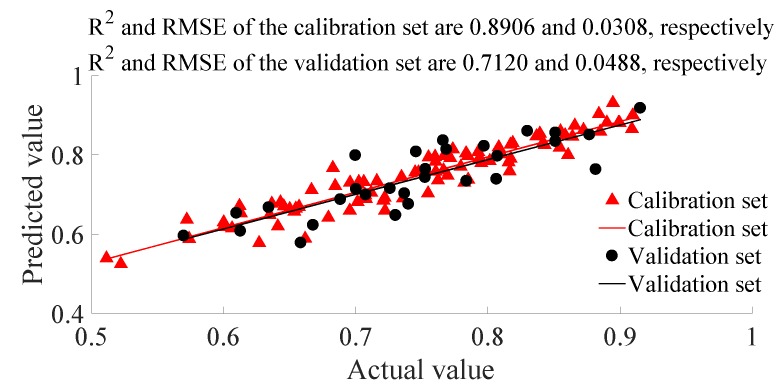
Pomelo water content PLSR detection result based on the SG + MSC data processed spectrum.

Due to SG and MSC being the superior data processing methods for the accurate analysis of detection data, to further check their accuracy improving effect, fourth-order seven-point SG followed by MSC was used. This data processing method showed an even better detection accuracy than SG or MSC alone, with the R^2^ and RMSE of the calibration set being 0.8906 and 0.0308, respectively, and the R^2^ and RMSE of the validation set being 0.7120 and 0.0488, respectively (Figure 4). Thus, SG + MSC was used for the spectrum data processing method in subsequent pomelo water content detection modeling work.

#### 3.1.3. Detection Based on Feature Selection Methods

**Figure 5:**
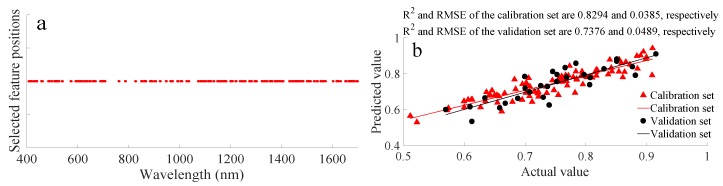
Pomelo water content PLSR detection based on SG + MSC data processing and GA feature selection: (**a**) corresponding wavelengths of selected features and (**b**) PLSR detection result based on SG + MSC + GA processed data.

Data for the whole spectral wavelength includes large amounts of information about pomelo, and it is important to determine which relevant/useful information should be kept and which irrelevant/redundant information should be removed to improve the detection accuracy. In this research, RCA, MI-SPA, GA, and PCA were used to select which wavelengths to ignore to reduce interference from redundant information and simplify the data structure. The pomelo water content PLSR detection results of the SG + MSC data processing method combined with different feature selection methods are shown in Table 2. GA and PCA provided better detection results than RCA and MI-SPA. During application, the PCA feature selection method always needed to map the spectral data to the stated dimension, then extract the first 118 principal components for modeling. The GA feature selection method, however, could directly extract the spectral data at different wavelengths for modeling. The number of features extracted using GA was 488 (Figure 5a), while the number of features extracted using PCA was only 118; however, 488 was an acceptable number and barely influenced the detection speed. Additionally, the R^2^ of the PLSR detection result based on SG + MSC with GA was better than with PCA for the validation set. Thus, GA was the optimal feature extraction method, with R^2^ and RMSE values for the calibration set of 0.8294 and 0.0385, respectively, and R^2^ and RMSE values for the validation set of 0.7376 and 0.0489, respectively. The pomelo water content PLSR detection result based on SG + MSC data processing and GA feature selection is also shown in Figure 5b.

### 3.2. Granulation Degree Detection

#### 3.2.1. LDA Classification Based on Raw Data

**Figure 6:**
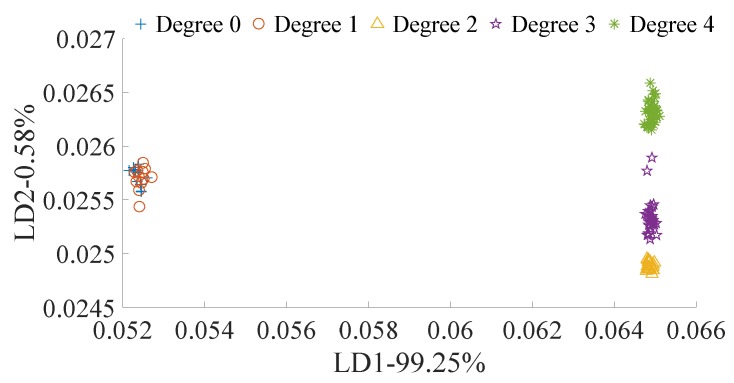
LDA granulation degree classification based on the spectral raw data.

According to the granulation statistical results, among all tested samples, 13 had a granulation degree of 0, 12 had a granulation degree of 1, 19 had a granulation degree of 2, 31 had a granulation degree of 3, and 44 had a granulation degree of 4. LDA was applied for the granulation degree classification, and the results are shown in Figure 6. Samples with degrees 0 and 1 were overlapped, and therefore could not be differentiated. The other granulation degree groups were differentiated completely.

#### 3.2.2. LDA Classification Based on Different Data Processing Methods

**Figure 7:**
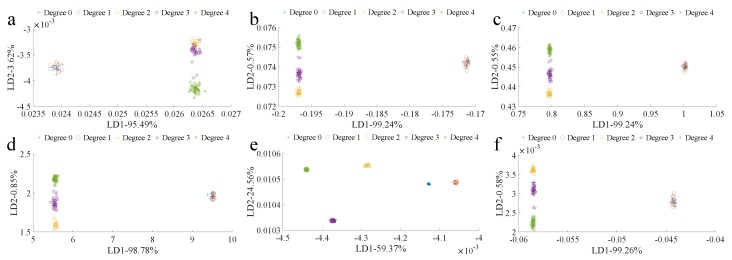
LDA granulation degree classification based on processing data using different methods:(**a**) FD, (**b**) SR, (**c**) LM, (**d**) IM, (**e**) SG, and (**f**) MSC.

Data processing methods (FD, SR, LM, IM, SG, and MSC) were applied with the aim of enlarging the differences between degrees to further improve the detection accuracy (Figure 7). The FD, SR, LM, IM, and MSC could not enlarge the difference between degrees 0 and 1. FD actually decreased the difference between degrees 3 and 4. However, the third-order five-point SG was able to enlarge the differences between degrees, while clustering the sample points in the same degree closer, dramatically improving the classification results. The granulation degree could be differentiated using LDA after SG processing, but calibration and validation are needed to prove that the good detection effect was not caused by overfitting.

#### 3.2.3. PLSR Detection Based on SG Processed Data

**Figure 8:**
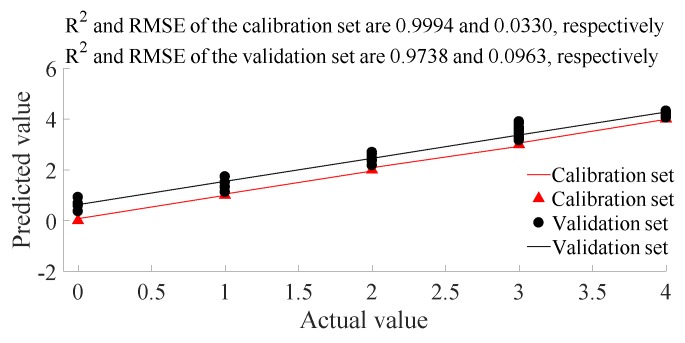
PLSR granulation degree detection based on SG processed spectral data.

Tofurthercheck the feasibility of nondestructive pomelo granulation detectionusing VIS/NIR spectroscopy, after separating the data into calibration and validation sets, the PLSR granulation degree detection results based on the SG processed spectral data are shown in Figure 8. The R^2^ values of both calibration and validation sets were larger than 0.97, and the RMSE values of both calibration and validation sets were less than 0.1, indicating an accurate detection result analysis. After taking the integer of the PLSR output, the classification accuracy of both the calibration and validation sets were 100%. Thus, it isfeasible to use VIS/NIR spectroscopy combined with an SG + PLSR modeling method to nondestructively detect the degree of granulation in pomelo since the detection speed and accuracywere satisfactory.

### 3.3. Relationship between Water Content and Granulation Degree

**Figure 9:**
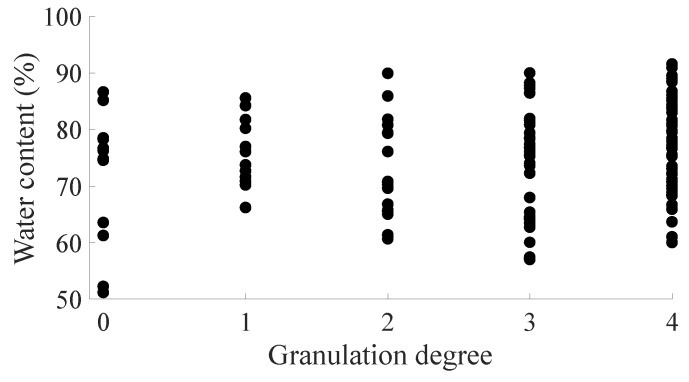
Relationship between the water content (%) and granulation degree of pomelo.

Previous research showed two opposite ideas regarding the relationship between water content and granulation of pomelo. Some supposed that the water content of pomelo flesh increased with the increase of granulation [4], while others suggested that the water content of pomelo flesh decreases with the increase of granulation [3]. However, these previous studies based their results on only 30 to 50 pomelo fruits and with different storage durations. To further check the relationship between water content and granulation, the results of this study were based on 119 pomelo samples and storage for 3 months (Figure 9). According to the distribution of data points, there was no apparent evidence to show a relationship between the water content and granulation. The statistical difference (*P* value) [33] between water content and granulation was 4.6747 × 10^−38^, indicatinga high certainty that there was no relationship between water content and granulation.

### 3.4. Discussion

From 2014 to 2018, the pomelo yield ofMeizhou (the major pomelo agricultural region in China) increased from 729.1 to 1000 thousand tons, but the price decreased from 5 to 3.7 Yuan/kg, which indicates a bottleneckfor the Chinese pomelo industry. One of the major reasonsfor market saturation is thatthe internal quality of pomelo iseasily influenced by planting conditions and can vary considerably during post-harvest, with no external indication of any internal differences. This is particularly true for “shatian” pomelo, which has a relatively high TSS (total soluble solids) content. For “shatian” pomelo, granulation andwater content are the most important parameters affectingtaste, and there is demand from the industryfor a nondestructive method to measure them. In this study, the water content and granulation of pomelo fruit were successfully and nondestructively detectedusing VIS/NIR spectroscopy.

The nondestructive detection of the internal quality of large thick peeled fruit has been largely neglected. Some studies showed the feasibility of VIS/NIR for the internal quality detection of large, thick-peeled fruit [20], but only for TSS, not for other quality indexes like water content or granulation. VIS/NIR is an efficient way to detect the water content of small fruit [34,35], but of unknown usefulness for detecting the water content of large, thick-peeled fruit like pomelo. On the other hand, as with pomelo, granulation is a major problem for oranges, and this has been extensively investigated [36]. Previously, it was proven that the detection of orange granulation could be accomplished using X-ray radiographs [37], but VIS/NIR has the advantage of cost, efficiency, safety, etc.overusing X-rays. Thus, this studyis an improvement over existing studies.

In this study, the water content was optimally detected using the SG + MSC + GA + PLSR method. After data preprocessing (SG + MSC) and feature selection (GA), even thoughthe R^2^ of the validation set only improved from 0.6928 to 0.7376,the R^2^ of the calibration set reduced from 0.9063 to 0.8294, but this was due to overfitting, and the validation result was more important (more closely represents the actual detection) when the calibration result was still good. In practical detection, the interference/noise may be larger and may cause a more serious overfitting problem. Thus, SG + MSC + CA is still suggested before modeling. SG +MSC could be quickly performed using a computer. GA took time for the modeling process operation; however, performing the practical detection process after all the features were set can be done quickly. Thus, SG + MSC + GA + PLSR is a feasible and practical method for thedetectionof pomelo water content. However, further improvement of the detection accuracy and the overfitting (the difference in R^2^ between calibration and validation sets) reductionwas still needed. Multi-sensor information fusion may be a feasible way to solve this problem in future research. The granulation degree could be reliably detected using SG + PLSR with the detectionaccuracy of the validation set being100%, and the number of features (under 1276) barely affecting the computation efficiency of PLSR due to the relatively simple structure of PLSR. Thus, there is no need to add a feature selection step to avoid an increase in the complexity of operation.

Feature combination is a complex issue. Generally, features with stronger relationshipswith the output values and that are detected at more sensitive spectral wavelengths should be selected. However, highly related and sensitive features might contain similar information. When this happens, a highly related feature combined with a feature with a lower relation may result ina better detection efficiency than combining two highly related features. Thus, the selected features are dispersedthroughout the whole experimental spectral wavelength using GA (Figure 5a).

Additionally, many pomeloindustrial expertssuggest that pomelo with good internal quality should be sold as fresh fruit, whileinferiorpomelo should be assigned to deep processing for things like pomelo peel candy production, fruit pulp production, essential oil extraction, flavone extraction, etc. The ability to perform this assignation relies on an accurate method for the nondestructive detection ofthe current internal quality. The method presented here for the detection of pomelo internal quality is accurate enough to provide the pomelo industry the ability to segregate fruit based on its internal quality. Using this method, fruit with a granulation degree of 0 and with high water content should be sold fresh. The peel and nongranulated flesh of low-water-content fruit with a granulation degree of 0 and 1, and the peel alone of fruit with granulation degrees 2, 3, and 4 can be assigned for deep processing.

## 4. Conclusions

The applicability of VIS/NIR spectroscopy for the nondestructive determination of internal water content and granulation degree of postharvest pomelo was studied in this research. CA was applied for singular sample elimination, which in turn greatly improved the PLSR water content detection accuracy. The pomelo water content PLSR detection results based on different spectral data processing methods (FD, SR, LM, IM, SG, and MSC) showed that SG + MSC was the optimal data processing method for the pomelo water content detection analysis. Pomelo water content PLSR detection results based on SG + MSC data processing and different feature extraction methods (RCA, MI-SPA, GA, and PCA) showed that GA was the optimal feature extraction method for the pomelo water content detection analysis. The LDA pomelo granulation degree classification results based on raw spectral data showed poor classification results for fruit with a granulation degree of 0 or 1. The LDA pomelo granulation degree classification results based on different spectral data processing methods (FD, SR, LM, IM, SG, and MSC) showed that SG was the optimal data processing method for the accurate detection of the granulation degree, and all granulation degrees were well-differentiated using this method. PLSR combined with SG data processing further proved that it is feasible to detect the pomelo internal granulation degree based on spectroscopy. Finally, our results suggest that the relationship between the pomelo water content and granulation was very weak (*p* value = 4.6747 × 10^−38^). The nondestructive detection method developed in this study is expected to be used to develop a real-time spectral detection device for the water content and granulation grading of stored pomelo before marketing.

## Figures and Tables

**Table 1:** PLSR pomelo water detection results after different data processing methods.

Data Processing Methods	NS	FD	SR	LM	IM	SG	MSC	RD
R^2^ at calibration set	90	0.9355	0.8815	0.8286	0.6247	0.9096	0.8974	0.9163
RMSEat calibration set		0.0273	0.0321	0.0386	0.0571	0.0280	0.0299	0.0270
R^2^ at validation set	29	0.4185	0.5638	0.4955	0.4480	0.7053	0.6998	0.6928
RMSE at validation set		0.0702	0.0647	0.0743	0.0690	0.0527	0.0500	0.0542

Note: NS—number of samples, FD—first derived method, SR—squareroot method, LM—logarithm method, IM—inverse method, SG—Savitzky–Golay method, MSC—multiplicative scatter correction method, and RD—raw data for comparison.

**Table 2:** PLSR pomelo water detection results combined with SG + MSC and different wavelength selection methods.

Selection Methods	Number of Features	Calibration Set (90 Samples)		Validation Set (29 Samples)	
		R^2^	RMSE	R^2^	RMSE
RCA	278	0.9872	0.0105	0.3885	0.0904
MI-SPA	990	0.9101	0.0279	0.6481	0.0592
GA	488	0.8294	0.0385	0.7376	0.0489
PCA	118	0.8906	0.0308	0.7120	0.0488
